# Lipid Control and Social Determinants: Their Association With LDL and Non‐HDL Cholesterol Goals in Older Adults—A Population‐Based Study

**DOI:** 10.1002/clc.70146

**Published:** 2025-05-07

**Authors:** Cristian Orlando Porras Bueno, Jesús Andrés Beltrán España, Carolina Murgueitio Guzmán, Cándida Diaz‐Brochero, Ángel Alberto García Peña

**Affiliations:** ^1^ Department of Internal Medicine Hospital Universitario San Ignacio, Pontificia Universidad Javeriana Bogotá Colombia

**Keywords:** cholesterol, cross‐sectional studies, heart disease risk factors, LDL, primary health care, risk assessment, social determinants of health

## Abstract

**Background:**

Social determinants of health (SDH) significantly influence cardiovascular outcomes; however, their role in achieving lipid profile goals in older adults remains underexplored. This study aimed to evaluate the association between SDH and nonachievement of LDL cholesterol (LDL‐C) and non‐HDL cholesterol (non‐HDL‐C) targets in Colombian adults aged ≥ 60 years.

**Methods:**

A cross‐sectional analysis was conducted using data from 1270 participants in the SABE Colombia 2015 study. Logistic regression was applied to estimate odds ratios (ORs) for not achieving LDL‐C and non‐HDL‐C goals on the basis of the Framingham, ASCVD 2013, and SCORE2 models.

**Results:**

Non achieving LDL‐C goals were associated with female sex, lack of affiliation with the health system and physical inactivity. Physical inactivity was associated with noncompliance on non‐HDL‐C goals, among others.

**Conclusions:**

SDH are strongly associated with poor lipid control. Addressing modifiable SDHs could enhance cardiovascular risk management in older adults.

## Summary

- Several social determinants of health are associated with poor lipid control.
- Female sex, lack of health system affiliation, and physical inactivity were associated with nonachievement of the LDL cholesterol goals.
- Physical inactivity was associated with noncompliance with the non‐HDL cholesterol goals.

## 1 Introduction

The ageing of the global population is a phenomenon in constant progression, with a rate of ageing that is faster than in the past [1, 2, 3]. Between 2015 and 2050, the percentage of the global population aged over 60 years is expected to nearly double, rising from 12% to 22% [1]. Furthermore, it is estimated that by 2050, 80% of older adults will reside in low‐ and middle‐income countries [1]. The incidence of cardiovascular disease (CVD) also increases significantly with age, reaching 75% in patients aged 60–79 years and 86% in those aged 80 years or older [4, 5].

The prevalence of dyslipidaemia is also higher among older adults, reaching, for instance, up to 76% in some European populations [4, 5, 6]. A meta‐analysis of eight primary prevention trials in individuals over 65 years of age with high cardiovascular risk (CVR) revealed a significant reduction in the risk of myocardial infarction (39.4%) and stroke (23.8%) with statin use compared with placebo [7]. However, the decision to use statins in older adults should be shared with the patient, taking into account individual goals and values, as well as factors such as frailty, estimated life expectancy, time to benefit, comorbidities, and patient preferences [4].

The World Health Organisation defines social determinants of health (SDH) as “the conditions in which people are born, grow, live, work, and age,” which can affect health, well‐being, and quality of life [8]. Previous research has documented the influence of SDH on CVD incidence, demonstrating that factors such as low socioeconomic status, reduced social support, and limited access to healthcare are associated with increased CVD risk and poorer health outcomes [9, 10]. In Latin America, studies have also explored the impact of SDH on CVD incidence, showing that CVR is exacerbated by socioenvironmental and economic conditions [11, 12]. However, no study has specifically focused on achieving LDL cholesterol (LDL‐C) and non‐HDL cholesterol (non‐HDL‐C) targets according to CVR, particularly in older adults.

The aim of this study was to analyze the relationships among SDH, the CVR profile, and LDL‐C control in older Colombian adults via data from the SABE Colombia Study [13].

## 2 Methods

### 2.1 Design, Context and Data Collection

This study represents a secondary analysis of a cross‐sectional survey involving 23,694 older adults derived from the 2015 SABE study [13]. This study was conducted as a multicenter project by the Pan American Health Organisation and was supported by the Epidemiological Office of Colombia's Ministry of National Health.

The survey focused on Colombians aged 60 and above, employing a combination of purposive and random sampling methods across urban and rural areas to ensure a representative sample of the older adult population. Data collection was conducted by trained personnel via standardized protocols for conducting face‒to‐face interviews and performing physical measurements to ensure data reliability.

Data processing and coding from the 2015 SABE study: A thorough review and validation were conducted after data collection to verify the quality and consistency of the information. Errors such as out‐of‐range values, logical inconsistencies, or duplicate records were corrected. Open‐ended responses were manually categorized into predefined codes, whereas closed‐ended responses were associated with numerical codes. All the information was standardized by assigning unique identifiers to the participants and normalizing the data according to the survey's variable dictionary.

Data digitization was consolidated into a storage system, depending on the design, either through manual input or direct entry from electronic devices. The data were subsequently organized and processed via statistical software such as SPSS, Stata, or R, which strictly adhered to the survey's variable dictionary and coding manual. Ethical approval for our secondary analysis from this study was granted by the institutional ethics and research committee (CIE‐1094‐23). Also, this study was in accordance with the last Declaration of Helsinki.

### 2.2 Operational Definition of Variables and Social Determinants of Health

According to the multilevel theoretical model of the health determinants proposed in the article from Ocampo et al., SDH can be divided into four levels: the first level includes biological factors and genetic flow, with age, sex, and race as examples of its variables; the second level includes lifestyle factors of the individual, such as being in a poverty economic situation or having chronic diseases; the third level refers to social and community network factors, such as marital status, violence in childhood, and origin, among others; and the fourth level includes socioeconomic, cultural, and environmental condition factors [14].

The LDL‐C goals for Framingham CVR categories are as follows: high risk < 70 mg/dL; moderate risk < 100 mg/dL; low risk < 116 mg/dL; ASCVD 2013 CVR categories: high risk < 70 mg/dL; intermediate risk and borderline risk < 100 mg/dL; low risk < 116 mg/dL; concerning SCORE2, for very high risk < 55 mg/dL; high risk < 70 mg/dL; moderate risk < 100 mg/dL; and low risk < 116 mg/dL [15, 16, 17]. Additionally, with respect to non‐HDL‐C goal achievement, it was measured only for the global SCORE2 according to the European guidelines recommendation [16], with a goal of < 85 mg/dL for very high risk, < 100 mg/dL for high risk, < 130 mg/dL for moderate risk, and < 150 mg/dL for low risk.

Sex was categorized as male or female (defined as the set of biological attributes that are associated with physical and physiological features), and marital status was classified as married (legally united in matrimony), cohabiting (living together without legal marriage), divorced (legally separated from a marriage), widowed (loss of a spouse), or single (without a legal or de facto partner). The health insurance scheme was defined according to affiliation with the healthcare system, grouped as contributory (self‐funded or employer‐funded contributions), subsidized (intended for vulnerable populations), exceptional (for specific groups, such as armed forces), special (particular schemes), or uninsured (without formal access to a healthcare system).

Racial identity was determined on the basis of participants’ self‐perception, encompassing the categories of indigenous, black, white, mixed‐race, other, or no response. The highest educational level achieved was classified from no formal education to postgraduate studies, including primary and secondary education (incomplete or complete), technical/technological training (with or without a degree), and university education (with or without a degree), with the option of no response.

The longest‐held occupation was assessed on the basis of the type of work performed, including categories such as private company employee (employed in a for‐profit organization), government employee (working in public institutions), rural laborer (working in agricultural or livestock activities), employer or business owner (self‐employed business owner), freelancer (self‐employed without a formal contract), volunteer (unpaid work), independent professional (autonomous professional practice), pieceworker (paid by specific output), domestic worker (paid domestic tasks), other occupation (not fitting into the aforementioned categories), or no response.

Body mass index (BMI) was calculated from measured weight and height and categorized into underweight, normal weight, overweight, and obese groups. Finally, the clinical comorbidities analyzed included hypertension, diabetes mellitus, and smoking status, which were recorded as present or absent.

### 2.3 Statistical Analysis

Categorical variables are presented as absolute numbers and percentages. Continuous variables are summarized as medians with 25th and 75th percentiles (interquartile ranges [IQRs]). The percentage of compliance with lipid profile goals was estimated according to the CVR categories according to Framingham, ASCVD 2013, and SCORE2 calibrated to Colombia [18]. SCORE2‐OP was calculated in patients aged 70 years or older without a conversion factor because this CVR scale is not externally validated in Colombia and was included with the SCORE2 in the global SCORE2 (SCORE2 and SCORE2‐OP).

Prevalence ratios (PRs) were calculated to identify which variables were associated with the nonachievement of LDL‐C goals according to the Framingham, ASCVD 2013, and global SCORE2 equations. The PRs reflect the frequency with which the nonattainment of the LDL‐C goal occurs in relation to certain characteristics. Additionally, to identify factors that increase the likelihood of not achieving the LDL‐C goal, a logistic regression analysis was conducted, estimating the odds ratios (ORs) with 95% confidence intervals (CIs) that were calculated. All the statistical comparisons were two‐tailed, and *p* < 0.05 was considered statistically significant. The calculations were carried out with the help of the statistical program StataCorp (2015) Stata Statistical Software: Release 14. StataCorp LP, College Station.

## 3 Results

**FIGURE 1:**
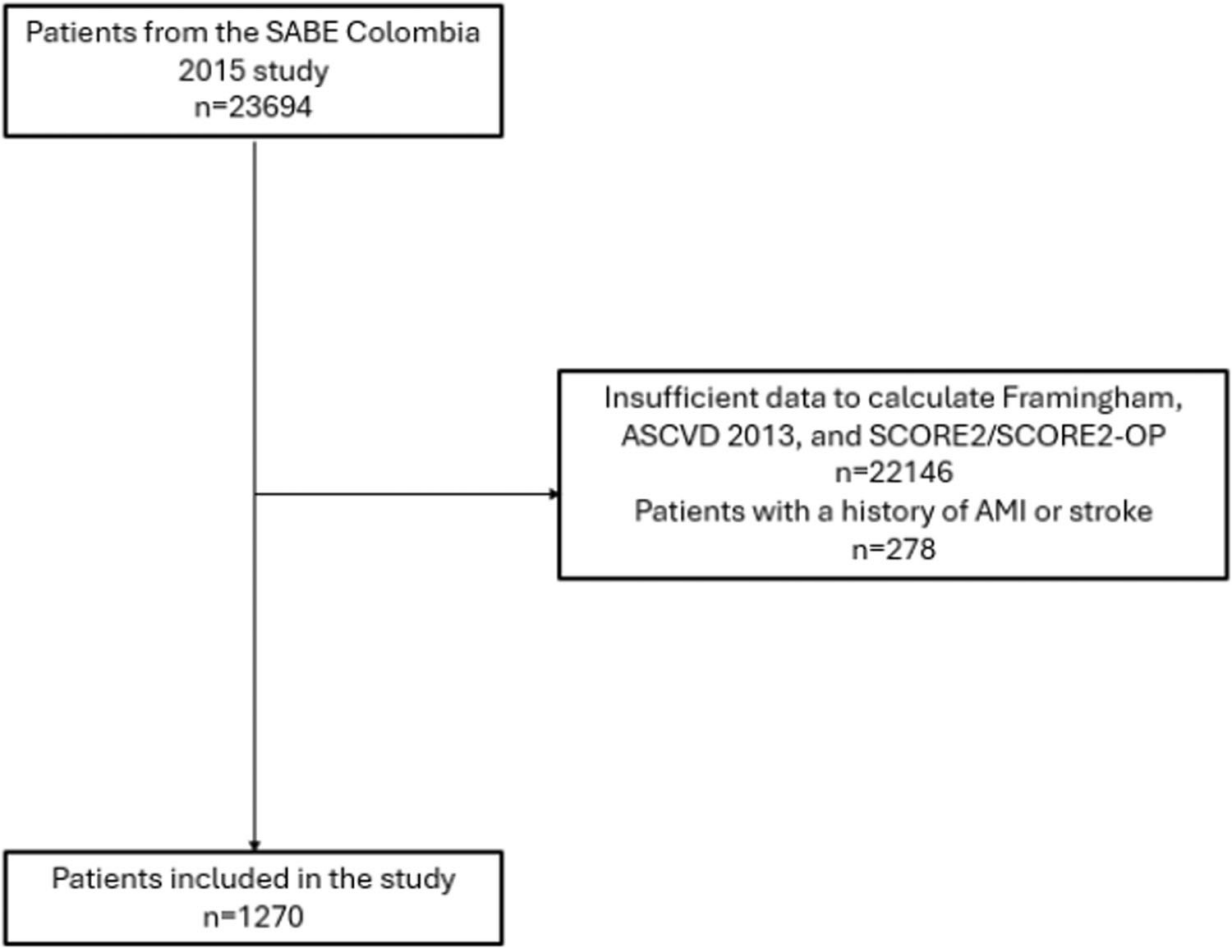
Study patient selection flowchart. AMI, Acute myocardial infarction.

Among the 23,694 patients aged 60 years or older in the SABE Colombia 2015 study, 1270 patients were included in our study: 22,146 were excluded because they had insufficient data to calculate their CVR according to Framingham, ASCVD 2013, and SCORE2 validated for Colombia [18] or insufficient data to estimate their CVR via the SCORE2‐OP. Moreover, 278 patients had a history of acute myocardial infarction or stroke, for which they were also excluded (Figure 1).

Among the 1270 patients, 60.79% (*n* = 772) were women, 41.97% (*n* = 533) were mixed race, 55.12% (*n* = 700) had a subsidized health regimen, 39.61% (*n* = 503) had incomplete primary school education, and 27.48% (*n* = 349) were employees of a company. In particular, 80.16% (*n* = 1018) did not perform moderate‐intensity physical activity at least three times a week. A complete description of the sociodemographic characteristics of this population is presented in Table 1.

**TABLE 1:** Sociodemographic characteristics of the population.

Characteristic	Male *n* = 498	Female *n* = 772	Total *n* = 1270
**Marital status**			
Married Cohabitation Divorced Widower Single	242 (48.59) 104 (20.88) 46 (9.23) 62 (12.45) 44 (8.83)	244 (31.60) 76 (9.84) 95 (12.30) 245 (31.73) 112 (14.50)	486 (38.27) 180 (14.17) 141 (11.10) 307 (24.18) 156 (12.28)
**Health regimen**			
Contributory Subsidized Exception Special Without affiliation	187 (37.55) 282 (56.62) 2 (0.40) 8 (1.60) 19 (3.81)	326 (42.23) 418 (54.14) 5 (0.64) 9 (1.16) 14 (1.81)	513 (40.39) 700 (55.12) 7 (0.55) 17 (1.34) 33 (2.60)
**Racial identity**			
Indigene Black White Mixed race Other Without answer	44 (8.85) 28 (5.62) 125 (25.10) 207 (41.56) 26 (5.22) 68 (13.65)	26 (3.37) 41 (5.31) 219 (28.34) 326 (42.23) 42 (5.44) 118 (15.28)	70 (5.51) 69 (5.43) 344 (27.09) 533 (41.97) 68 (5.35) 186 (14.65)
**Highest educational level achieved**			
None Incomplete primary Complete primary Incomplete secondary Complete secondary Technician/Technologist without title Technician/Technologist with title University student without a degree University graduate Postgraduate degree holder Without answer	80 (16.06) 192 (38.55) 86 (17.23) 76 (15.26) 30 (60.02) 2 (0.40) 9 (1.81) 4 (0.80) 13 (2.61) 3 (0.60) 3 (0.60)	113 (14.64) 311 (40.28) 158 (20.47) 80 (10.36) 39 (5.05) 2 (0.26) 24 (3.19) 4 (0.52) 11 (1.43) 6 (0.77) 4 (0.52)	213 (16.77) 503 (39.61) 244 (19.21) 156 (12.28) 69 (5.43) 4 (0.31) 33 (2.60) 8 (0.63) 24 (1.89) 9 (0.71) 7 (0.55)
**Work performed longer**			
Worker/Employee private company Laborer/Government Employee Rural laborer or field laborer Employer or owner Freelancer Volunteer Independent professional Pieceworker Domestic employee Other Without answer	158 (31.73) 48 (9.64) 158 (31.73) 7 (1.41) 98 (19.68) 2 (0.40) 3 (0.60) 8 (1.61) 2 (0.40) 13 (2.61) 1 (0.20)	191 (24.74) 56 (7.25) 44 (5.70) 5 (0.65) 136 (17.62) 6 (0.77) 4 (0.52) 6 (0.77) 157 (20.34) 12 (1.55) 155 (20.08)	349 (27.48) 104 (8.19) 202 (15.91) 12 (0.94) 234 (18.43) 8 (0.63) 7 (0.55) 14 (1.10) 159 (12.52) 25 (1.97) 156 (12.28)
**Hypertension**			
No Yes	287 (57.63) 209 (42.37)	325 (42.10) 446 (57.90)	612 (48.30) 655 (51.70)
**Diabetes mellitus**			
No Yes	439 (88.15) 59 (11.85)	637 (82.51) 135 (17.49)	1076 (84.72) 194 (15.28)
**Smoker**			
No Yes	428 (85.94) 70 (14.06)	716 (92.75) 56 (7.25)	1 144 (90.08) 126 (9.92)

Compliance with the LDL‐C goals according to the different risk categories across the three prediction models is summarized in Table 2.

**TABLE 2:** Compliance with the LDL and non‐HDL cholesterol goals according to CVR categories among Framingham and ASCVD 2013 calibrated to Colombia and global SCORE2 (SCORE2/SCORE2‐OP).

CVR equation	LDL‐C goal achievement (%)	Non‐HDL‐C goal achievement (%)
**Framingham**		
High risk Moderate risk Low risk	0.72 15.77 41.49	Does not apply Does not apply Does not apply
**ASCVD 2013**		
High risk Intermedium risk Borderline risk Low risk	3.93 20.34 20.94 34.11	Does not apply Does not apply Does not apply Does not apply
**Global SCORE2**		
Very high risk High risk Moderate risk Low risk	1.01 1.51 20.97 Without data^a^	3.78 5.54 39.78 —

Abbreviations: CVR, Cardiovascular risk; LDL‐C , LDL cholesterol; Non‐HDL‐C, Non‐HDL cholesterol.

a No patient was categorized as low risk according to the global SCORE2.

### 3.1 Associations Between the CVR Equations and Not Attaining the LDL‐C Goals

After the percentages of LDL‐C goal achievement were obtained according to the Framingham and ASCVD 2013 equations adjusted for Colombia [18], as well as the global SCORE 2 (SCORE2/SCORE2‐OP), PRs were calculated to estimate the variables associated with not achieving LDL‐C goals according to each of the risk categories from the three CVR equations. Additionally, a logistic regression with ORs estimation was conducted to identify the factors associated with nonachievement, which are described below.

With respect to the associations of PRs with non‐LDL‐C goal achievement, the female sex, according to Framingham and ASCVD 2013, was associated with nonattainment of LDL‐C goals. Additionally, the widowed status and having been a domestic worker were associated with the three CVR equations; being without affiliation with the national health system according to the Framingham and ASCVD 2013 equations and physical inactivity were associated with an increased risk of not achieving the LDL‐C goal according to the global SCORE2. The PRs found with their respective CIs are described in Table 3.

**TABLE 3:** Prevalence ratios and their associations with nonachievement in LDL‐C goals.

Variable	Framingham: PR (95% CI)	ASCVD2013: PR (95% CI)	Global SCORE2: PR (95% CI)
**Sex**			
Female	**1.19 (1.11–1.29)** a	**1.15 (1.07–1.23)** a	1.01 (0.98–1.03)
**Marital status**			
Widowed	**1.09 (1.01–1.19)** a	**1.12 (1.04–1.20)** a	**1.02 (1.001–1.05)** a
**Health regimen**			
Without affiliation	**1.26 (1.10–1.45)** a	**1.19 (1.04–1.37)** a	Without data to estimate PR.
**Highest educational level achieved**			
Complete primary	**0.87 (0.78–0.98)** a	**0.89 (0.81–0.99)** a	0.99 (0.95–1.02)
**Work performed longer**			
Domestic employee	**1.12 (1.005–1.24)** a	**1.14 (1.04–1.25)** a	**1.03 (1.0001–1.07)** a
**Physical activity three times a week**			
No	1.05 (0.96–1.15)	1.06 (0.97–1.15)	**1.05 (1.01–1.09)** *

Abbreviations: CI, confidence interval; PR, prevalence ratio.

a Highlighted and bold results obtained a *p* value < 0.05.

### 3.2 PRs Between Prediction Models and Nonattainment of the Non‐HDL‐C Goal According to the Global SCORE2 (SCORE2/SCORE2‐OP)

No association between sex and nonachievement of the non‐HDL‐C goal according to the global SCORE2 was found (PR 1, 95% CI 0.96–1.04). Additionally, despite no association being found between marital status categories and nonattainment of the non‐HDL‐C goal, being widowed tended to favor an increase in the nonattainment of the non‐HDL‐C goal (PR 1.04, 95% CI 0.99–1.08).

With respect to affiliation status according to our national health system, being in the subsidized regimen was associated with an increase in the likelihood of not achieving the non‐HDL‐C goal (PR 1.04, 95% CI 1.01–1.09, *p* value = 0.01) compared with being in the contributory regimen. With respect to race and the highest educational level achieved in the global SCORE2 and nonattainment of the non‐HDL‐C goal, no associations were found.

Additionally, similar to the association found between physical inactivity and nonattainment of LDL‐C in the global SCORE2, physical inactivity was associated with an increase in the likelihood of not achieving the non‐HDL‐C goal (PR 1.06, 95% CI 1.007–1.119, *p* value = 0.026).

### 3.3 ORs Between Prediction Models and Nonattainment of LDL‐C Goals

With respect to the associations of ORs with non‐LDL‐C goal attainment, the female sex, widowed marital status, and complete primary status as the highest educational level achieved were associated with non‐LDL‐C achievement according to the Framingham and ASCVD 2013 prediction models. Additionally, being black according to the racial identity only for the ASCVD 2013 and physical inactivity was associated with an increased risk of not achieving the LDL‐C goal according to the global SCORE2. The ORs found in the bivariable analysis with their respective CIs are described in Supporting Information S1: Table 1.

### 3.4 ORs Between Prediction Models and Non‐HDL‐C Goals

The associations of ORs with non‐HDL‐C goal achievement were analyzed only for the global SCORE2, which revealed that belonging to the subsidized health regime (OR 1.6, 95% CI 1.12–2.41, *p* value = 0.01) was associated with not meeting non‐HDL‐C goals; likewise, having worked as a laborer or government employee and physical inactivity were associated with not meeting non‐HDL‐C goals (OR 2.7, 95% CI 1.07–7.21; OR 1.7, 95% CI 1.13–2.62, respectively).

### 3.5 Multivariate Analysis of ORs

In addition, logistic regression with multivariate analysis was performed across the three prediction models (Framingham, ASCVD 2013 and global SCORE2). The ORs found in the three multivariable analyzes with their respective CIs are described in Table 4.

**TABLE 4:** Multivariable analysis models with odds ratios for nonattainment of the LDL‐C goal.

Variable	Framingham: OR (95% CI)	ASCVD2013: OR (95% CI)	Global SCORE2: OR (95% CI)
**Sex**			
Female	**1.80 (1.35–2.40)** a	**1.74 (1.32–2.30)**	Not included in the model
**Health regimen**			
Without affiliation Subsidized	**3.01 (1.01–8.98)** a	Not included in the model	**1 (empty)**
	1.27 (0.96–1.67)		**2.37 (1.30 – 4.34)** a
**Physical activity three times a week**			
No	Not included in the model	Not included in the model	**2.60 (1.45–4.67)** a

Abbreviations: CI, confidence interval; OR, odds ratio.

a Highlighted and bold results obtained a *p* value < 0.05.

## 4 Discussion

Our study examined the relationship between SDHs and the nonattainment of LDL‐C goals among adults aged 60 years and older, utilizing three widely used cardiovascular risk calculators. Failure to achieve LDL‐C goals was associated with female sex, lack of health system affiliation, and physical inactivity. Targeting modifiable SDHs may improve cardiovascular risk management in this population.

Similar to our results, Goodman et al. found in the DYSIS cross‐sectional Canadian study that among high‐risk patients according to the 2006 Canadian Cardiovascular Society recommendations, being a woman was associated with an increase in the likelihood of not attaining the LDL‐C goal (adjusted OR 1.32, 95% CI 1.08–1.61) [19], a finding close to that of the study from Gavina et al. [20], which also revealed that being a woman was associated with a decrease in the likelihood of achieving the LDL‐C goal (hazard ratio [HR] 0.78, 95% CI 0.73–0.82). In addition, in a similar way, the results from the study of Schultz et al. support the sex difference described above because being male was associated with achievement of the LDL‐C goal (OR 1.27, 95% CI 1.12–1.46) among the patients from her study [21].

Furthermore, Schultz et al. [21] also revealed that older individuals, as well as individuals who were compliant with their statin therapy, were more likely to achieve their LDL‐C goal (OR 1.02, 95% CI 1.01–1.04; OR 5.18, 95% CI 3.29–8.17, respectively) [21]. The latter is similar to the findings of Rodriguez et al., who also reported that being on statin therapy was associated with an increase in the likelihood of attaining the LDL‐C goal (OR 2.92, 95% CI 2.30–3.71) [22].

Nevertheless, in a cross‐sectional study by Alwhaibi et al. where an association between statin adherence and LDL‐C goal attainment was explored among patients with a mean age of 58.8 years who had type 2 diabetes and dyslipidemia, no association between adherence to statin therapy and LDL‐C goal attainment was found (adjusted OR 0.72, 95% CI 0.97–1.66) [23].

The differences found between the mentioned studies [21, 22, 23] could reflect the fact that although adherence to statins favors the achievement of LDL‐C goals, they are not the only relevant factor for the same goal, and other variables could be associated with the achievement of LDL‐C goals. Unfortunately, in our study, we cannot explore the association between statin therapy and compliance because no information about statin prescription or compliance was provided in the 2015 SABE Colombia study.

Moreover, in the study performed by Rodrigues et al. where LDL‐C goal attainment (according to CVR using the Framingham risk score) was evaluated in hypertensive patients with a median age of 59.9 ± 11.1, they reported that diabetes and CVD were associated with an increase in the probability of not attaining the LDL‐C goal in their study (OR 2.94, 95% CI 2.16–4; OR 4.51, 95% CI 2.99–6.80, respectively) [24]. Despite these findings, diabetes is a variable included in the Framingham CVR risk calculator, and its behavior corresponds to that of a variable that enhances the CVR; thus, collinearity is likely present in this finding.

In addition, Nguyen et al. [25] investigated the prevalence and factors associated with the lack of achievement of LDL‐c goals in older type 2 diabetes mellitus patients with a very high risk of CV diseases. This study revealed that being obese increases the likelihood of not attaining the LDL‐C goal (OR 2.33, 95% CI 1.13–4.81), whereas being on high‐intensity statin therapy could decrease this probability (OR 0.03, 95% CI 0.01–0.05) [25]. However, notably, age, sex, educational level, and comorbidities were not associated with not attaining LDL‐C goals.

In our study, although being obese was not associated with not attaining the LDL‐C goal, being in the underweight category when CVR risk was estimated according to Framingham was associated with a decrease in the likelihood of not achieving the LDL‐C goal (OR 0.48, 95% CI 0.24–0.96), which, together with the findings from Nguyen et al. [25] suggests that an abnormal BMI could act as a variable associated with reaching the LDL‐C goal. Together, our findings and those previously reported and discussed demonstrate that some SDH are linked to goal noncompliance, highlighting the necessity of implementing primary prevention strategies in the older adult population that target SDH and other modifiable factors that affect CVR among older adults.

In addition, our study has several limitations: a lower external validity of the data obtained for the global SCORE2, which included the SCORE2 and SCORE2‐OP prediction models, resulted from the fact that SCORE2‐OP was calculated without a conversion factor because this prediction model was not externally validated in Colombia. This condition could have eventually led to an overestimation of CVR in older adults aged 70 years or more. Furthermore, because of this, it is possible that no patients were found in the low‐risk category when analyzing the global SCORE2 risk categories. Second, owing to the cross‐sectional study design, the presence of associations between variables through PRs can be explored, which does not allow testing a causality hypothesis since it is impossible to determine the temporal sequence between them. Moreover, logistic regression with bivariable and multivariable analyzes to estimate ORs was performed to mitigate these limitations.

Finally, we are unable to completely rule out biases that arise from survey research, including selection bias, nonresponse bias, memory bias, and information bias. However, the qualitative variables, such as age, sex, smoking status, diabetes or hypertension status, and smoking status, that were utilized to determine the CVR in the three prediction models were simple to respond to and recall. Similarly, measurements of the quantitative variables employed, including HDL cholesterol, total cholesterol, and systolic blood pressure, were taken at the time of the survey.

## 5 Conclusion

With respect to the achievement of LDL‐C goals in older adults and their relationship with SDH, our study identified several associations among their four levels, including being female, not being affiliated with the health regimen, and physical inactivity, among others, highlighting the fact that some of these factors are modifiable, indicating the necessity of implementing social interventions across these social determinants to improve them and eventually attain the LDL‐C and non‐HDL‐C lipid profile goals.

## Ethics Statement

This study was approved by the institutional ethics committee (CIE‐1094‐23).

## Conflicts of Interest

The authors declare no conflicts of interest.

## Supporting information

Supplementary Table 1. Odds ratios for non‐attainment of LDL‐C goal.

## Data Availability

The data that support the findings of this study are available from the corresponding author upon reasonable request.
