# Supplementary material for: Lipid Control and Social Determinants: Their Association With LDL and Non‐HDL Cholesterol Goals in Older Adults—A Population‐Based Study
Source: Clin Cardiol. 2025 May 7;48(5):e70146. doi: 10.1002/clc.70146 (PMC12058642; doi:10.1002/clc.70146)
Supplement: Supplementary file 1 — Supplementary Table 1. Odds ratios for non‐attainment of LDL‐C goal. [file CLC-48-e70146-s001.docx]

**Supplementary material**

**Supplementary Table 1. Odds ratios for non-attainment of LDL-C goal.**

| **Variable** | **Framingham: OR (95% CI)** | **ASCVD2013: OR (95% CI)** | **Global SCORE2: OR (95% CI)** |
| --- | --- | --- | --- |
| **Gender**  Female | **1.86 (1.45 – 2.39)*** | **1.78 (1.37 – 2.32)*** | 1.34 (0.77 – 2.34) |
| **Marital status**  Widowed | **1.41 (1.02 – 1.96)*** | **1.69 (1.18 – 2.41)*** | 2.37 (0.95 – 5.93) |
| **Health regimen**  Without affiliation | **3.19 (1.10 – 9.24)*** | 2.64 (0.91 – 7.65) | Without data to estimate OR. |
| **Highest educational level achieved**  Complete primary  Complete secondary school | **0.63 (0.41 – 0.95)***  0.77 (0.41 - 1.41) | **0.62 (0.39-0.87)***  0.60 (0.32 - 1.14) | 0.75 (0.26 – 2.16  **0.22 (0.07 – 0.66)*** |
| **Work performed longer**  Domestic employee  Independent professional | 1.54 (0.99 – 2.39)  1.09 (0.20 - 5.71) | **1.85 (1.15 – 2.98)***  0.94 (0.18 - 4.97) | 2.48 (0.83 – 7.35)  **0.16 (0.02 – 0.87)*** |
| **Physical activity three times a week**  No | 1.19 (0.88 – 1.61) | 1.26 (0.92 – 1.73) | **2.89 (1.63 – 5.12)*** |

**CI = Confidence interval. OR = Odds ratio. *Highlighted and bold results obtained a *p* value < 0.05.**
